# 13 million years of seafloor spreading throughout the Red Sea Basin

**DOI:** 10.1038/s41467-021-22586-2

**Published:** 2021-04-23

**Authors:** Nico Augustin, Froukje M. van der Zwan, Colin W. Devey, Bryndís Brandsdóttir

**Affiliations:** 1 GEOMAR Helmholtz Centre for Ocean Research, Kiel, Germany; 2 King Abdullah University of Science and Technology (KAUST), Thuwal, Saudi Arabia; 3 Institute of Earth Sciences, Science Institute, University of Iceland, Reykjavík, Iceland

**Keywords:** Geodynamics, Structural geology, Tectonics

## Abstract

The crustal and tectonic structure of the Red Sea and especially the maximum northward extent of the (ultra)slow Red Sea spreading centre has been debated—mainly due to a lack of detailed data. Here, we use a compilation of earthquake and vertical gravity gradient data together with high-resolution bathymetry to show that ocean spreading is occurring throughout the entire basin and is similar in style to that at other (ultra)slow spreading mid-ocean ridges globally, with only one first-order offset along the axis. Off-axis traces of axial volcanic highs, typical features of (ultra)slow-spreading ridges, are clearly visible in gravity data although buried under thick salt and sediments. This allows us to define a minimum off-axis extent of oceanic crust of <55 km off the coast along the complete basin. Hence, the Red Sea is a mature ocean basin in which spreading began along its entire length 13 Ma ago.

## Abstract (web summary)

Here, based on earthquake data, vertical gravity gradient data and high-resolution bathymetry, the authors show that the Red Sea is not in transition from rifting to spreading as previously proposed. They instead suggest it to be a mature ocean basin in which continuous seafloor spreading began quasi-instantaneously along its entire length around 13 Ma ago.

## Introduction

**Fig. 1:**
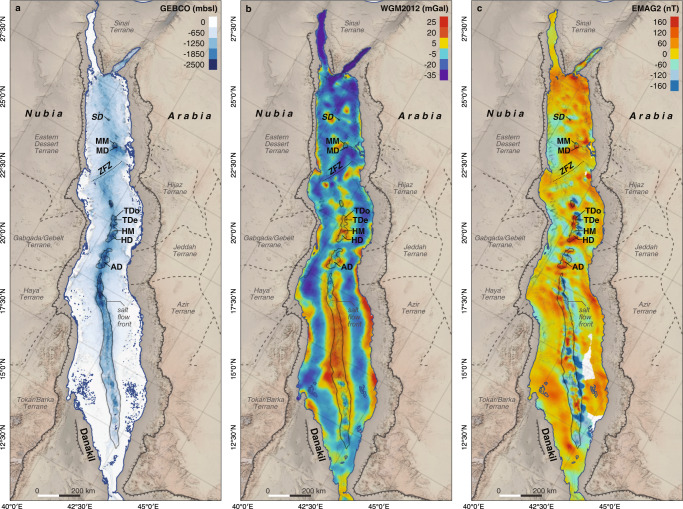
Overview maps of the Red Sea. **a** 15-arc-seconds gridded GEBCO 2020 bathymetry (resolution of 435 m at 20°N). **b** Free-air gravity anomalies from global WGM2012 dataset, 1-arc-min resolution (1.7 km at 20°N). **c** Magnetic anomalies from global EMAG2v3 dataset, 2-arc-min resolution (3.4 km at 20°N). The position of bathymetric deeps and large axial volcanoes discussed in this work are marked. SD = Shaban Deep, MM = Mabahiss Mons, MD = Mabahiss Deep, ZFZ = Zabargad fracture zone, TDo = Thetis Dome, TDe = Thetis Deep, HM = Hatiba Mons, HD = Hatiba Deep, AD = Aswad Dome. All maps were generated with QGIS, projection WGS84.

**Fig. 2:**
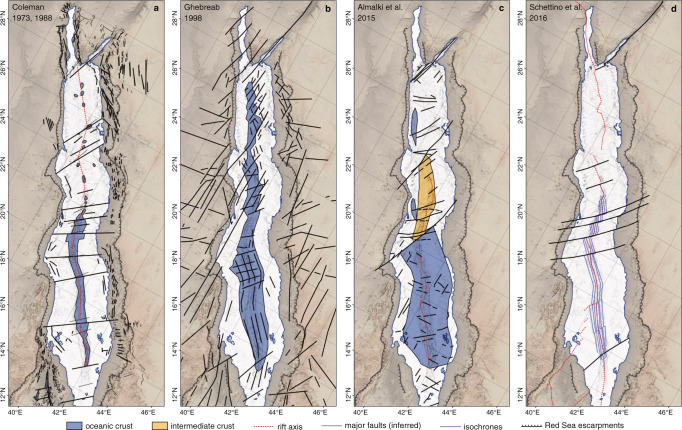
Diversity of tectonic models of the Red Sea. **a** Initial models suggested continuous spreading in the south grading northwards into discrete spreading nodes separated by continental crust^4,5^. **b** Model allowing for oceanic crust along the entire basin^8^. **c** Model with large areas of intermediate crust in the central Red Sea^6^. **d** Model limiting oceanic spreading to areas where magnetic isochrones were identified^7^. Common for all models is the significant amount of large transform faults and fault zones across the entire basin.

The Red Sea (Fig. 1) is one of Earth’s youngest ocean basins and the type-locality to examine continental rifting and the transition to ocean spreading^1–3^. Yet, despite its geological importance, tectonic models developed for the Red Sea are highly diverse and debated (Fig. 2), making it difficult to determine what role this type-locality can play in our understanding of continental breakup. Common structural features of all previously proposed models are the presence of the Zabargad fracture zone (ZFZ) and fault and lineament patterns of varying complexity both to explain anomalies in geophysical data and to accommodate the variably complicated tectonic processes thought necessary to create the observed bathymetry^4–8^ (Fig. 2). The locations of these faults are highly variable in terms of extent and orientation and often contradictory between the different models (Fig. 2). The models also differ greatly in the amount and distribution of oceanic crust proposed to occur along the Red Sea, varying from continuous ocean crust in the southern Red Sea and scattered nodes of ocean spreading within continental crust towards the North^1,4–7,9^ (Fig. 2a), to continuous ocean spreading throughout the basin^3,8^ (Fig. 2b) with various alternatives, including large areas with intermediate crust^6^ (Fig. 2c), between these extremes. As a result, the proposed age and extent of continental breakup varies in the models between continental rift stages in the process of breakup to a basin in full-ocean spreading.

A major barrier to advancing our understanding of Red Sea structure are the large amounts of sediment, including extensive salt deposits, in the basin. This cover makes direct observation of the underlying crust in many cases impossible, and it introduces uncertainties that lead to non-unique interpretations of geophysical signals (in particular, gravity and magnetics). In the case of magnetics, eruption beneath a salt and sediment blanket suppresses the formation of a normal volcanic upper crust, which may produce unusual magnetic signatures^10–12^. Several other regions of the global mid-ocean ridge system which are buried beneath km-thick layers of sediments are known to exhibit an absence of interpretable magnetic anomalies. Examples are the Guaymas Basin (Gulf of California)^10^, Escabana Trough (southern Gorda Ridge)^10^, Middle Valley (Juan de Fuca Ridge)^10^, the northern Labrador Sea^11^ or the eastern Gakkel Ridge^13^. The dikes or sills that form the upper part of the crust under a thick sediment-cover, cool slower and crystallise larger mineral grains with a lower specific magnetic remanence or different polarity than extrusive rocks of a similar composition^10,14^. These dikes and sills contribute less to the total magnetisation of the crust, which results in a lack of standard magnetic anomalies due to weaker or incoherent magnetisation^12^. Furthermore, the magnetic signals of seafloor basalts can be considerably reduced by hydrothermal alteration^14^. The possibility of extensive hydrothermal alteration along sediment-buried rifts—causing the breakdown of magnetite below the sediment blankets—has been extensively discussed^3,10^ and provides a comparatively simple explanation for magnetic quiet zones at sediment (and salt) covered, active mid-ocean ridges. To further compound the problem, oblique spreading along short en-échelon segments creates magnetic source blocks that, in ship-based or air-borne surveys, are too small to be resolved into clear magnetic anomaly stripes, generating instead areas with seemingly low-intensity magnetisation and little coherent magnetic structure. This effect is especially strong during the early stages of ocean spreading^11,15^. Whatever mechanism or combination of processes leads in the end to the weak magnetic signatures beneath sediment covers, it undoubtedly makes the picking of datable anomalies challenging^12^ which may in turn influence the interpretation of other data such as gravity^2,3^. To advance, we need to use data less influenced by the presence of a thick salt and sediment cover.

**Fig. 3:**
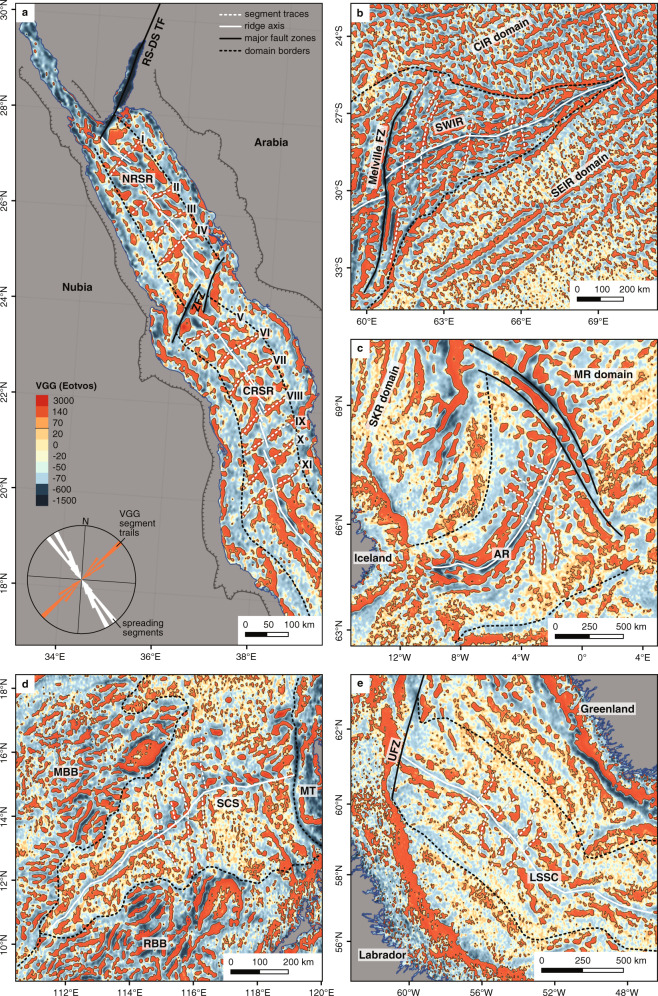
Off-axis segmentation trails in slow-spreading ocean rifts revealed by vertical gravity gradient data. **a** In the widely salt- and sediment-covered Red Sea Basin, the vertical gravity gradient data reveal axis-perpendicular segmentation trails north of 20°N. The identified trails (dashed white lines) are numbered from north to south by roman numbers. The rose diagram shows the average direction of rift-axis segments (white) vs. the average direction of segmentation trails (orange). RS-DS-TF = Red Sea–Dead Sea transform fault, NRSR = northern Red Sea Rift, ZFZ = Zabargad fracture zone, CRSR = central Red Sea Rift. **b** The non-sediment buried Southwest Indian Ridge (SWIR) within the Central Indian Ocean domain (CIR) and the Southeast Indian Ocean domain (CEIR); FZ = fracture zone. **c** Aegir Ridge (AR), buried under <1 km thick sediment cover^18^ (SKR = Southern Kolbeinsey Ridge, MR = Mohns Ridge). **d** South China Sea (SCS) spreading centre covered by 1–2 km sediment^19^ (MBB = Macclesfield Bank Block, RBB = Reed Bank Block, MT = Manila Trench). **e** The Labrador Sea Spreading Centre (LSSC) buried under 2 km sediments^20^ (UFZ = Ungava Fault Zone). The quantile colour-key is the same for all maps, and the segmentation trails are well visible at ≥70 E (eotvos), which marks the maximum value of the full-width at half-maximum (FWHM) mean range of the global vertical gravity gradient dataset.

In this work, we integrate vertical gravity gradient (VGG) data that reveal crustal structures also beneath thick sediment packs^16,17^ with bathymetric and seismic data^17^ (Fig. 3) to study the tectonic structure and the nature of the Red Sea crust and to present a straightforward tectonic concept for the development of this young ocean basin during the last 13 Myr (million years).

## Results and discussion

### Buried rift segmentation visible in VGG data

**Fig. 4:**
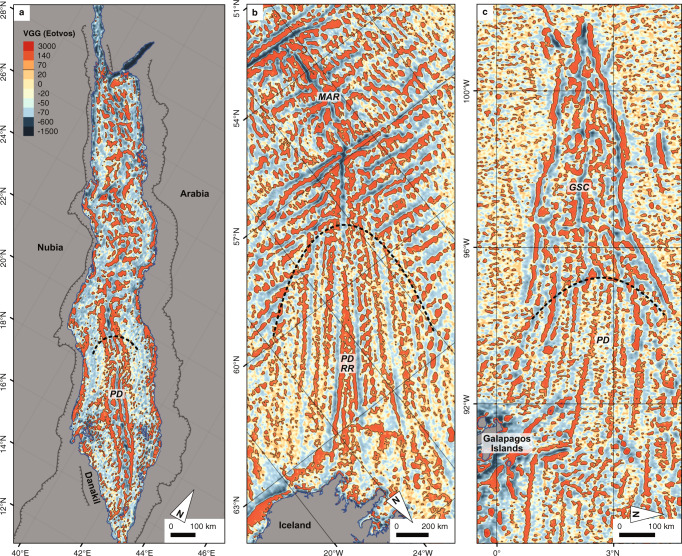
Vertical gravity gradient patterns of slow-spreading mid-ocean ridges approaching mantle plumes. **a** Red Sea Rift towards the Afar plume. **b** The Mid-Atlantic Ridge (MAR) and Reykjanes Ridge (RR) towards the Iceland Plume. **c** The Galapagos Spreading Centre (GSC) towards the Galapagos Hotspot. All three examples show that rift-oblique to rift-perpendicular off-axis segmentation trails are vanishing with increasing plume influence and the vertical gravity gradient patterns become rift-parallel. The views are rotated to match the orientation of the Red Sea map (panel **a**) with the plume always located towards the bottom of each map (see north-indicators). PD = Plume Domain, roughly indicated by the black dashed line. The colour-key is the same as in Fig. 3 for all panels.

**Fig. 5:**
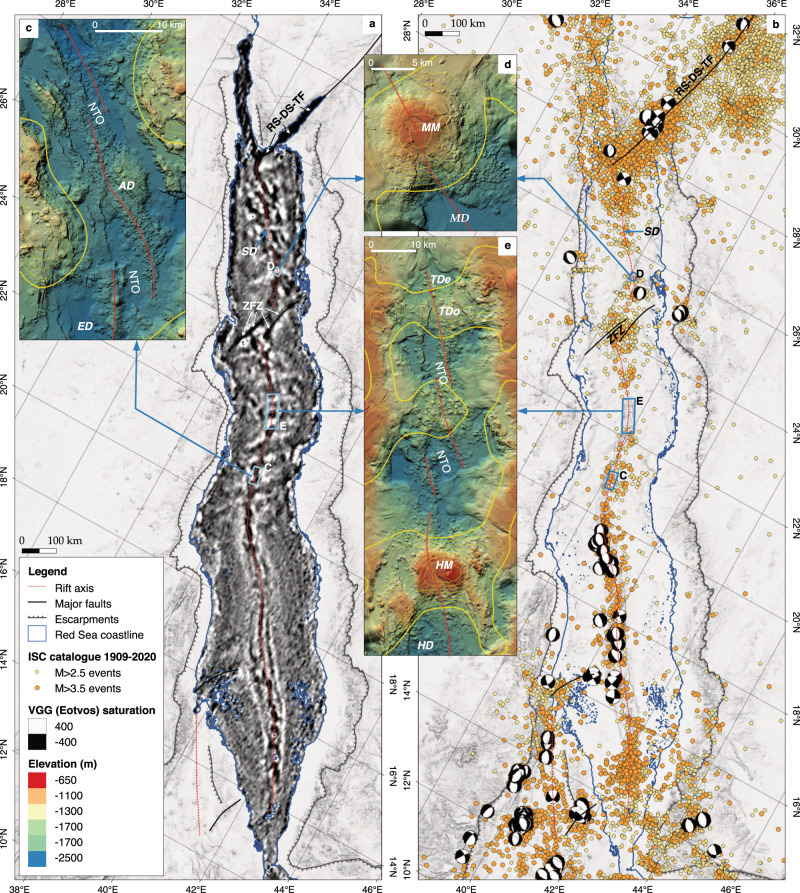
Vertical gravity gradient, seismicity and bathymetry of the Red Sea Rift. **a** The vertical gravity gradient (VGG)^16^ data reveal hidden structures under the salt and sediment blankets in the Red Sea. Rift-perpendicular, stripy positive anomalies represent off-axis segmentation trails, caused by along-axis crustal thickness variations^26^, known from slow-spreading mid-ocean ridges elsewhere^17^ (see also Figs. 3 and 6). Shaban Deep (SD) represents the northernmost site where a salt-free rift axis was observed. First-order transform offsets are represented by strong negative VGG anomalies and are only visible in the Zabargad fracture zone (ZFZ) and the Red Sea–Dead Sea transform fault (RS-DS-TF). **b** The position and intensity of earthquake epicentres M > 2.5 between 1906 and 2020 (International Seismological Centre, ISC-GEM Earthquake Catalogue^31^) is delineating the plate boundaries and areas of transform faults in the Red Sea. Global CMT moment tensor solutions^31^ indicate strike-slip for the northern boundary of the Danakil block as well as for the Red Sea–Dead Sea transform fault in the Gulf of Aqaba (symbols are drawn on top of epicentre positions). **c** High-resolution ship bathymetry showing along axis details of the ocean crust in area north of Erba Deep (ED) around the Aswad Dome (AD). **d** Bathymetric details of the prominent Mabahiss Mons volcano (MM) and the Mabahiss Deep (MD) in the northern Red Sea. **e** Bathymetry of the Red Sea Rift from Thetis Deep (TDe) to Hatiba Deep (HD) with the large axial volcanic centres of the Thetis Dome (TDo) and Hatiba Mons volcano (HM). The large dome volcanoes and axial highs are the recent magmatic centres of the segmentation trails seen in VGG and where eventually seismic quiet zones are present. Yellow lines in panels (**c**–**e**) represent VGG contours ≥70 E (100 E steps), red line = rift axis, NTO = second-order non-transform offset (for details see text); C, D and E indicate the position of the detail maps.

Long-lived axial segmentation and discontinuities produce along-axis variations in crustal structure whose trails, on other ridges, can be traced up to 1000 km and over 30 Ma (million years ago) off-axis^17^. These segmentation trails are an integral part of (slow-spreading) oceanic crust and can be observed where, e.g. the ultra-slow-spreading Southwest Indian Ridge opens between the Central Indian Ridge and Southeast Indian Ridge domains (Fig. 3), but more importantly, also in VGG data from now inactive slow to medium-spreading ocean rifts that are buried under km-thick sediments such as at the North Atlantic Aegir Ridge (≈1 km sediment)^18^, in the South China Sea (1–2 km sediment)^19^ or the Labrador Sea (2 km sediment^20^, Fig. 3). In all the cases (with and without sediments), the VGG data reveal similar patterns that are exclusive to slow-spreading ocean crust^16,17^. The consistent magnitude (>70 E, eotvos) of the rift-perpendicular VGG anomalies in all the examples confirms that it is not significantly modified by a sediment cover (Figs. 3 and 4). Similar rift-perpendicular positive VGG anomalies are observed in the central and northern Red Sea (Figs. 3 and 5), where they coincide with axial volcanoes and axial highs (e.g. Mabahiss Mons and Hatiba Mons, Fig. 5c, e) and thus indicating segmentation trails below the salt and sediment cover^21^.

South of 20 °N, where the axis is continuously exposed, we do not see along-axis variations in water depth or volcanic activity and the off-axis rift-perpendicular trails are absent. This transition also corresponds to a significant shallowing of the axis. This probably reflects an increasing influence of the Afar plume on the ridge, increasing magma supply and so reducing or eliminating the magmatic focussing which produces the VGG trails. We note that the VGG patterns in the Red Sea south of 20 °N much more closely resemble those observed along, e.g. the Reykjanes Ridge^17,22^ and Galapagos Spreading Centre^16^—other plume-influenced slow-spreading ridges (Fig. 4). Excess magma production presumably affects the dynamics of ridge segmentation, diminishing the magmatic focussing towards segment centres and creating generally thicker crust, leading to the observed differences in VGG anomalies at these locations^17,23,24^.

### Non-transform offsets and the position of the rift axis

In previous models, most prominent rift-perpendicular gravity lows (Fig. 1b) were interpreted as being related to major, ridge-crossing fault zones^4–8^, often proposed to connect terrane borders of the Nubian and Arabian shields (Fig. 2) across the Red Sea Basin. Latest geomorphological works^21,25^, however, provided no support for the presence of large cross-cutting (transform) faults. No first-order ridge offsets^26^ or the typical transform valleys have been observed in any available bathymetric data of the Red Sea Rift. This could be because transform offsets and their valleys have been overflown and thus hidden under salt and sediments without any bathymetric expression at the seafloor. But other (ultra)slow-spreading mid-ocean ridges, including those covered by sediments, show that large oceanic transform faults are represented in free air gravity and VGG data by strong, narrow and negative rift-oblique or rift-perpendicular anomalies (−600 to −2000 E, Fig. 3), probably caused by, e.g. intense fracturing and significantly reduced crustal thickness^16,26,27^. In the Red Sea, such VGG signatures are only seen at the Dead Sea transform fault throughout the Gulf of Aqaba and the rift-oblique ZFZ at around 24 °N^16,21^ (Figs. 3 and 5a). GLORIA side-scan data revealed seafloor-flow patterns towards the ZFZ that suggest a possible salt and sediment infilled transform valley in this area^28^, confirming that the inflow of evaporites and sediment does not alter the VGG signal significantly.

In regions not blanketed by slumped salt and sediment, the high-resolution bathymetry along the Red Sea axis reveals a slow-spreading mid-ocean ridge morphology with a well-defined axial valley. Second-order (unfrequently third-order) right-lateral non-transform offsets^21,25^ (NTO, see also Fig. 5c, e) are expressed as en-échelon-like rift jumps of less than 10 km. This type of ridge segmentation is typical for (ultra)slow-spreading ridges and explained by an irregular along-axis melt supply towards the axial lithosphere, which is focussed to the segment centres^17,26,27^. The segment ends are typically characterised by less magmatism, NTOs, exposure of deeper crustal rocks or even oceanic core complexes, although the latter have not yet been identified along the Red Sea Rift^21^.

The distribution of earthquakes along the rift reveals both the positions of major transform faults and outlines the location of the spreading axis^29,30^. Seismic data provided by the ISC database^31–34^ clearly show intense seismic activity along the Red Sea–Dead Sea transform fault, in the area of the ZFZ and towards the Danakil Rift in the southern Red Sea. Furthermore, they show the presence of a central zone of focussed seismic activity along the entire length of the Red Sea, including the northern Red Sea (Fig. 5b). In regions of this earthquake zone where basement is exposed, bathymetric data show a mid-ocean-ridge-like morphology^3,21^ and sampling yielded normal mid-ocean ridge basalts^3,35,36^. The magnitude range, recurrence frequency and spatial density of earthquakes along the Red Sea axis is similar to those seen on other (ultra)slow-spreading ridge axes^30,37,38^. This includes areas with higher seismic activity and low recent volcanism^21^ (e.g. Aswad Dome, Fig. 4b, c) and seismically quieter areas (such as the Hatiba-Thetis area, with Thetis Dome and Hatiba Mons (22–23 °N) and the Mabahiss area with Mabahiss Mons (25–26 °N)^21^, see also insets (c–e) in Fig. 5), which can be explained by, geologically recent, higher volcanic activity^21,39^, resulting in less-brittle tectonic accommodation due to increasing influence of ductile magmatic processes in taking up the spreading strain. Taking further into account that the catalogues of seismic events only cover a limited time window (a few decades), we conclude that these areas represent a stage of the volcanic cycle that is marked by seismic quietness after an active phase, supported by the larger amount of recent lava flows in these areas^21,39^. With the exception of local and temporal seismic swarms from volcanic centres (e.g. 85 °E Gakkel Ridge in 1999^40^) that mark the beginning of volcanic cycles^41^, significantly lower seismic activity at volcanically active segment centres and large axial volcanoes has been also reported from the (ultra)slow-spreading Gakkel and Southwest Indian Ridge^37,38^.

Sparse station coverage means that earthquake source mechanisms are relatively few along the rift—those that are available indicate normal faulting on the axis^31,42^. Predominantly, left-lateral strike-slip focal mechanisms are seen along the Red Sea–Dead Sea transform fault and at the boundary between the Danakil microplate and the Nubian plate^43^ (NE of the Gulf of Zula at 16 °N, Fig. 4b). No focal mechanisms could be determined for the Zabargad fracture zone—the nearest solutions are normal faulting at the ridge axis (close to Mabahiss Deep) and the Red Sea escarpment on land.

The lack of typical transform fault signatures in the VVG data combined with the bathymetric and seismic data all suggest that the entire Red Sea axis is experiencing spreading, offset only by numerous second-order NTOs and the ZFZ.

### Extent and age of seafloor spreading

**Fig. 6:**
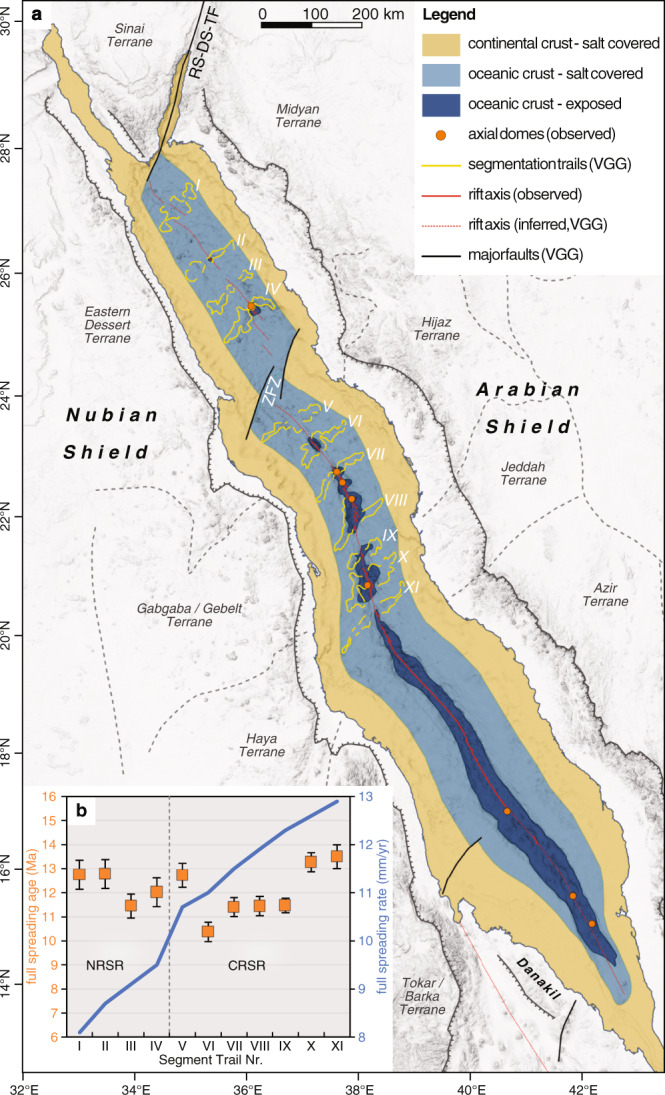
Resulting tectonic model for the Red Sea Rift. **a** The tectonic model of the Red Sea Rift is the result of combined observations from vertical gravity gradient (VGG) data, seismic activity and latest bathymetric maps and favours a continuous ocean crust along the entire length of the basin that is buried in large parts under salt and sediment flows (see legend, oceanic crust—salt covered). The only right-lateral, first-order offset of the rift axis is located in a significant fault zone at about 24°N (ZFZ = Zabargad fracture zone). The rift axis is further systematically offset by second-order non-transform offsets at segment ends, but no other first-order offsets (i.e. transform faults) can be observed along the rift axis. Off-axis segmentation trails identified from VGG represent mid-ocean ridge axial segmentation and mark the minimum extend of the oceanic crust. They are numbered from north to south (for details see text). **b** Plotted spreading ages of the rift-perpendicular segmentation trails show ages of up to 12.8 Myr in the northern Red Sea Rift (NRSR) and 13.5 Myr in the central Red Sea Rift (CRSR), averaging at 12.1 Myr (orange squares; error bars represent minimum and maximum range of calculated ages, see also Table 1). The full spreading rates are plotted by the blue line as listed in Table 1 and discussed in the text. RS-DS-TF = Red Sea–Dead Sea transform fault.

Making the simplest assumption that, in places where we observe the VGG signal for ridge-segmentation-trails, oceanic lithosphere will be present (even if it is not exposed on the seafloor due to the sediment overburden)^17^, we can use their ridge-perpendicular extents to provide an indication of the minimum extent of oceanic crust underneath the salt and sediment coverage in the Red Sea. We identified 11 segmentation trails between 20 °N and 27 °N (dashed lines in Fig. 3a). The overall length of individual segmentation trails varies from 103 km at 27 °N in the northern Red Sea (VGG ridge I; Figs. 3a, 6 and Table 1) to 174 km at 20.4 °N in the Central Red Sea (VGG ridge XI; Figs. 3a, 6 and Table 1). The trails extend up to <55 km of the shoreline. Using full spreading rates of 8.1 ± 0.4 mm/yr and 12.9 ± 0.4 mm/yr at these latitudes, respectively^43–45^, suggests the trails mark between at least 12.7 ± 0.6 and 13.5 ± 0.5 Myr of ocean spreading (Table 1 and inset in Fig. 6), not only in the southern and central Red Sea but also to the north of the ZFZ. This implies a significantly earlier start of seafloor spreading throughout the entire Red Sea than proposed by most other models for either the southern RSR (~5 Ma, inferred from magnetic anomalies^46–48^) or, more importantly, the northern RSR, which had previously been suggested to be still in its rifting phase. That the Northern Red Sea is experiencing spreading is in line with direct observations of oceanic basement exposed in slump-windows through the salt deposits, e.g. at Shaban and Mabahiss Deep (for locations, see Fig. 1). Basaltic samples taken there have the compositions of typical tholeiitic mid-ocean ridge basalt formed from asthenospheric decompression melting^3,35,36^, unlike the more alkaline, small-volume melts generally found in continental rifts^49^. Tectonically, the presence of oceanic crust along the entire Red Sea Rift is also consistent with the general lack of extensive continental thinning observed around the basin^50^, as separation without thinning requires that new lithosphere is produced, i.e. ocean spreading. These ages are only a minimum estimate, based on the visibility of segmentation traces in the VGG data and seafloor spreading could have started earlier. Studies from the Farasan Islands in the southernmost Red Sea suggest that even older oceanic crust of >20 Myr^51^ may be present there, which would imply either an earlier episode of opening of the southern Red Sea or an overall older age for oceanic spreading along the entire Red Sea. In both cases the whole Red Sea is currently undergoing oceanic spreading and has been for much longer than previously thought.

**Table 1:** Spatial characteristics of identified off-axis segmentation trails along the Red Sea Rift.

Off-axis segment trail no.	Centre lat (°N)	Centre long (°E)	Western extent (km)	Eastern extent (km)	Total length (km)	Obliquity index (deg)	Full spreading rate^44^ (mm/yr)	Spreading age (Myr)
I	27.0	34.7	48	55	103	101	8.1 ± 0.4	12.7 ± 0.6
II	26.2	35.3	58	53	111	82	8.7 ± 0.4	12.8 ± 0.6
III	25.8	35.7	68	36	104	94	9.1 ± 0.4	11.5 ± 0.5
IV	25.4	36.1	86	28	114	96	9.5 ± 0.4	12.0 ± 0.6
V	23.6	36.9	78	58	136	88	10.7 ± 0.4	12.7 ± 0.5
VI	23.2	37.2	40	74	114	103	11.0 ± 0.4	10.4 ± 0.4
VII	22.7	37.6	67	64	131	105	11.5 ± 0.4	11.4 ± 0.4
VIII	22.0	37.9	72	64	136	105	11.9 ± 0.4	11.4 ± 0.4
IX	21.4	38.1	58	83	141	99	12.3 ± 0.4	11.5 ± 0.3
X	20.9	38.2	93	74	167	107	12.6 ± 0.4	13.3 ± 0.4
XI	20.4	38.3	99	75	174	105	12.9 ± 0.4	13.5 ± 0.5

Spreading ages are calculated from full spreading rate and the overall length of the segment trails, regardless of eventual asymmetries and thus present a lower estimate. Obliquity Index describes the difference of the average segment trails orientation to the orientation of Red Sea spreading segments (obliquity index of 90 means perpendicular), thus the stronger the deviation to 90°, the more the length of the trails are underestimated (see also insets in Figs. 3a and 6). The spreading rates are taken for the given coordinates from the MORVEL database^44^ for reproducibility reasons, but the results are comparable to numbers given by other authors^43,45^.

We conclude that the Red Sea axis is completely underlain by oceanic crust as far north as the Red Sea–Dead Sea transform fault, which marks the northern bound of spreading. Our geological model of the Red Sea Rift has a simple tectonic structure (Fig. 6) which matches those of other global (ultra)slow-spreading ridges with extensive NTO ridge segmentation. Significant along-axis crustal thickness variations and the absence of large transform faults along the long stretches of these ridges is not atypical. Even when large proportions of the oceanic crust are covered by salt blankets and sediment flows, VGG data reveal hidden, off-axis segmentation trails along the central and northern Red Sea Rift, resembling VGG data at slow to medium spreading ridges in other oceans. Our model proposes continuous seafloor spreading in the entire Red Sea Basin that started all along the Red Sea Rift at least 12–13 Ma. This more than doubles the age of the oceanic crust so far assumed for the Red Sea. The calculated minimum age of spreading initiation based on these VGG ridges spans a relatively narrow range, suggesting that opening of the Red Sea was quasi-instantaneous and that the present-day Red Sea Rift is fully mature. This means that, in contrast to many models and studies over the last decades, the Red Sea is not an ocean in the transition between rifting and spreading but completed its rifting phase up to 8 Myr earlier than previously thought.

## Data Availability

GEBCO gridded global ocean bathymetry (Figs. 1, 2, 5 and 6) is available from https://www.gebco.net/data_and_products/gridded_bathymetry_data/, the WGM2012 global gravity model (Fig. 1b) from https://bgi.obs-mip.fr/data-products/grids-and-models/wgm2012-global-model/ and the EMAG2 earth magnetic anomaly grid (Fig. 1c) is available from https://www.ngdc.noaa.gov/geomag/emag2.html. Cross-blended hypsometric tints (Figs. 1 and 2) from Natural Earth https://www.naturalearthdata.com/downloads/10m-raster-data/10m-cross-blend-hypso/. Gridded vertical gravity gradient data (Figs. 3, 4 and 5a) are available from https://topex.ucsd.edu/grav_outreach/index.html#links_rel or directly from ftp://topex.ucsd.edu/pub/global_grav_1min/. Earthquake catalogues (Fig. 5b) are available from the International Seismological Centre http://www.isc.ac.uk. High-resolution bathymetry grids (Fig. 5c–e) are hosted on the Pangaea data repository under https://doi.pangaea.de/10.1594/PANGAEA.860374 and https://doi.pangaea.de/10.1594/PANGAEA.912178. Spreading rates between Nubian and Arabian plates are obtained (for the given coordinates in Table 1) from the MORVEL and NNR-MORVEL56 plate velocity estimates available at http://geoscience.wisc.edu/~chuck/MORVEL/motionframe_mrvl.html. All relevant data are also available from the authors.
